# The Festival of Healing

**Published:** 1920-12-25

**Authors:** 


### THE MATRONS' AND SISTERS' DEPARTMENT.

### THE FESTIVAL OF HEALING.

Christmas, by common consent, is the great festival of the hospitals, of all who dedicate themselves to the relief of suffering. The quickened sense of the Master's coming and continued presence among us which is set stirring in all hearts, however careless, by the Christmas bells, leads naturally to an impulse towards deeds of mercy and kindliness. Every one remembers, with desire to comfort and relieve, the sick and sorrowful at Christmastide.

Chief of all it is the nurses' festival. It is they who form the main-spring on which the joyous keeping of the festival among the sick is seen to turn; theirs the happy gift which changed in the years which are over even the shambles of the clearing stations to sanctuaries, and made of the great war hospitals such joyous temples of thanksgiving as have led many thousands now keeping holiday at home to look back on their war Christmases with awe and wonder. This gift, developed in hospital and never lost, has its origin in the origin of the nursing profession. It is by virtue of the Christmas secret that the true nursing sister is what she is. She radiates the light which shines in darkness, and makes manifest the truth revealed in the first Christmas song. It is as the mirror of God's love to erring suffering mortals that the nursing sister finds her place in the nation. This is her hold on rich and poor alike, quite irrespective of creed or forms of worship. If the infinite compassion be not reflected in her, however faintly, her work runs to waste, and her vocation becomes a mere weariness of the flesh.

In the new ideals which fill the collective mind of the nursing profession, it can be noted by those who look below the surface that the image of the light which shines in darkness has its sure place. It is not one of the things we talk about in conferences or committees. Englishwomen are very reticent about the things in life which are of real importance, though they will argue hotly and with persistence a question of procedure or etiquette. Yet the lives of the women who count in nursing politics are surely linked with God, and the ultimate aim of every training-school which has achieved distinction is so to polish the mind mirror in the nurses of this generation that they may reflect that love which heals and gladdens mankind.

Regarded from this point of view, every modern aspiration, every educational advance, every long-delayed reform has its part in lending nurses the power to flash back the rays of light. Has not the dreary, monotonous round of duties performed mechanically till head and heart were sick proved in times past the greatest of all impediments to the clear shining of the light? The human energies have been dulled past recovery by stupid or greedy authorities, and the spark of Divine fire within has often died down never to re-kindle. Generations of Heaven-inspired nurses have been sacrificed by the strain of duties over-long performed till they became material, pleasure-loving, self-seeking, or simply machines.

What, after all, is the aim of a perfected nursing education, if it be not to rub off the crust of ignorance and indifference and so develop the faculties that every action of the nurse may reflect not only the science, not only the skill, but the love from which true service springs? Slowly, one by one, the impediments are being removed which hinder nurses in the exercise of their vocation. All reforms have their birth in a new consciousness of evils which are marring the mind or distorting the aims. It is no mere desire to make things easy from the bodily point of view which is inspiring our nursing reformers. It is the penetrating wisdom which notes where and why the heart is getting warped. Unless the mirror be well and truly ground, unless it be turned towards the sun, it is vain to concentrate on the decorations of the frame—it can but reflect a blurred image of the light.

On this Christmas Day, which preludes the birth of a new system for the nursing profession, we pause to give thanks for the light shining in darkness, conscious that here is the germ of the nurse's vocation, the nurse's place in the heart of the nation.

